# Evaluation of retinal pigment epithelium changes in serous pigment epithelial detachment in age-related macular degeneration

**DOI:** 10.1038/s41598-021-82563-z

**Published:** 2021-02-02

**Authors:** Masahiro Miura, Shuichi Makita, Yoshiaki Yasuno, Takuya Iwasaki, Shinnosuke Azuma, Toshihiro Mino, Tatsuo Yamaguchi

**Affiliations:** 1 Department of Ophthalmology, Tokyo Medical University, Ibaraki Medical Center, 3-20-1 Chuo, Ami, Inashiki, Ibaraki 300395 Japan; 2 Computational Optics Group, University of Tsukuba, Tsukuba, Japan; 3 Topcon Corporation, Tokyo, Japan

**Keywords:** Translational research, Macular degeneration

## Abstract

The purpose of this study was to quantitatively evaluate retinal pigment epithelium (RPE) changes in serous pigment epithelial detachment (PED) among patients with age-related macular degeneration by means of prototype multi-contrast optical coherence tomography (OCT), which is capable of simultaneous collection of OCT angiography, polarization-sensitive OCT, and standard OCT images. We evaluated 26 eyes of 21 patients with serous PED. RPE-melanin OCT images were calculated from the multi-contrast OCT dataset and compared with near-infrared autofluorescence images. An active RPE lesion was defined as an area of thickened RPE-melanin (≥ 70 μm; RPE_70_) on RPE-melanin OCT. Each PED area was divided into peak and slope regions. RPE_70_ area ratios were compared with the maximum PED height, PED area, PED volume, and slope area ratio (area of slope region/area of whole PED). RPE-melanin OCT images were consistent with near-infrared autofluorescence images. The RPE_70_ area ratio in the slope region was significantly negatively correlated with the slope area ratio. Development of active RPE lesions in the slope region was correlated with the PED configuration. Multi-contrast OCT is useful for objective evaluation of changes in the RPE in patients with age-related macular degeneration.

## Introduction

Age-related macular degeneration (AMD) is a leading cause of vision loss in elderly adults^1^. Alterations of the retinal pigment epithelium (RPE) are an important sign of AMD^2–4^. Pigment epithelial detachment (PED) is a common finding in patients with AMD^5^. In eyes with a PED, especially those with a drusenoid PED, RPE cells show various responses, including sloughing, shedding, hypertrophy, and intraretinal migration^6–9^. These RPE changes are presumed to be important indicators of RPE function^7^. Therefore, clinical evaluation of RPE changes in PED is crucial for the clinical management of AMD.

Clinical autofluorescence (AF) imaging is widely used to evaluate RPE changes in AMD^8,10–12^. AF signals in short-wavelength AF (SW-AF; excitation 488 nm) imaging are presumed to originate from lipofuscin or melanolipofuscin^13^, while AF signals in near-infrared AF (NIR-AF; excitation 785 nm) imaging are presumed to originate from melanin or melanolipofuscin^14–16^. Simultaneous hyper-AF lesions in both SW-AF and NIR-AF are presumed to arise from an increased concentration of melanolipofuscin in RPE cells^12^, as well as RPE dysmorphia (greater cell height) and intraretinal RPE migration^8^. Although clinical AF imaging is widely used to evaluate RPE changes, the absence of topographic information remains an important limitation of AF imaging.

Intensity-based optical coherence tomography (i.e., standard OCT) is another essential clinical tool for evaluation of RPE changes^6,7,17^. Some intraretinal hyperreflective foci in standard OCT images have been attributed to intraretinal RPE migration^6–8,18^, which might be the origin of hyper-AF lesions^8^. If RPE cells are lost because of RPE migration, one would expect hypo-AF in the RPE layer^18^. One class of thickened RPE-Bruch’s membrane band in standard OCT might represent RPE dysmorphia and stacked RPE cells^7,19^. Standard OCT can provide important information concerning RPE changes; however, the evaluation of RPE cells using standard OCT is limited by the lack of specific contrast to RPE cells, which impedes direct comparisons with AF images.

Polarization-sensitive OCT is a functional extension of OCT technology that permits advanced tissue differentiation in retinal disease^20,21^. Multiple scattered lights from melanin in the RPE induce depolarization or scrambled polarization^22^. Comprehensive evaluation of RPE changes in AMD can be performed using a combination of polarization-sensitive OCT and AF imaging^8,23,24^. However, the evaluation of RPE changes by polarization-sensitive OCT has some limitations, including a lack of discriminability between melanin in the RPE (RPE-melanin) and melanin in the choroid. In projection images captured by polarization-sensitive OCT, RPE changes are obscured by depolarization involving choroidal melanin^25^. To overcome this limitation, we developed an automatic highlighting algorithm for RPE-melanin using multi-contrast OCT (MC-OCT)^26^, which enables simultaneous collection of OCT angiography, polarization-sensitive OCT, and standard OCT images^27–29^. From the MC-OCT dataset, we derived RPE–melanin-specific contrast OCT (RPE-melanin OCT) images, which we used to evaluate RPE changes^26^. In our previous studies of patients with AMD^26^ and patients with Vogt–Koyanagi–Harada disease^25^, we confirmed similarities between NIR-AF images and RPE-melanin OCT projection images. Thus, the combination of RPE-melanin OCT and AF imaging might be useful for evaluation of RPE changes in PED. In this study, we evaluated RPE changes in serous PEDs by means of AF and MC-OCT imaging. We also analyzed the relationships of RPE changes with PED parameters.

## Results

**Figure 1:**
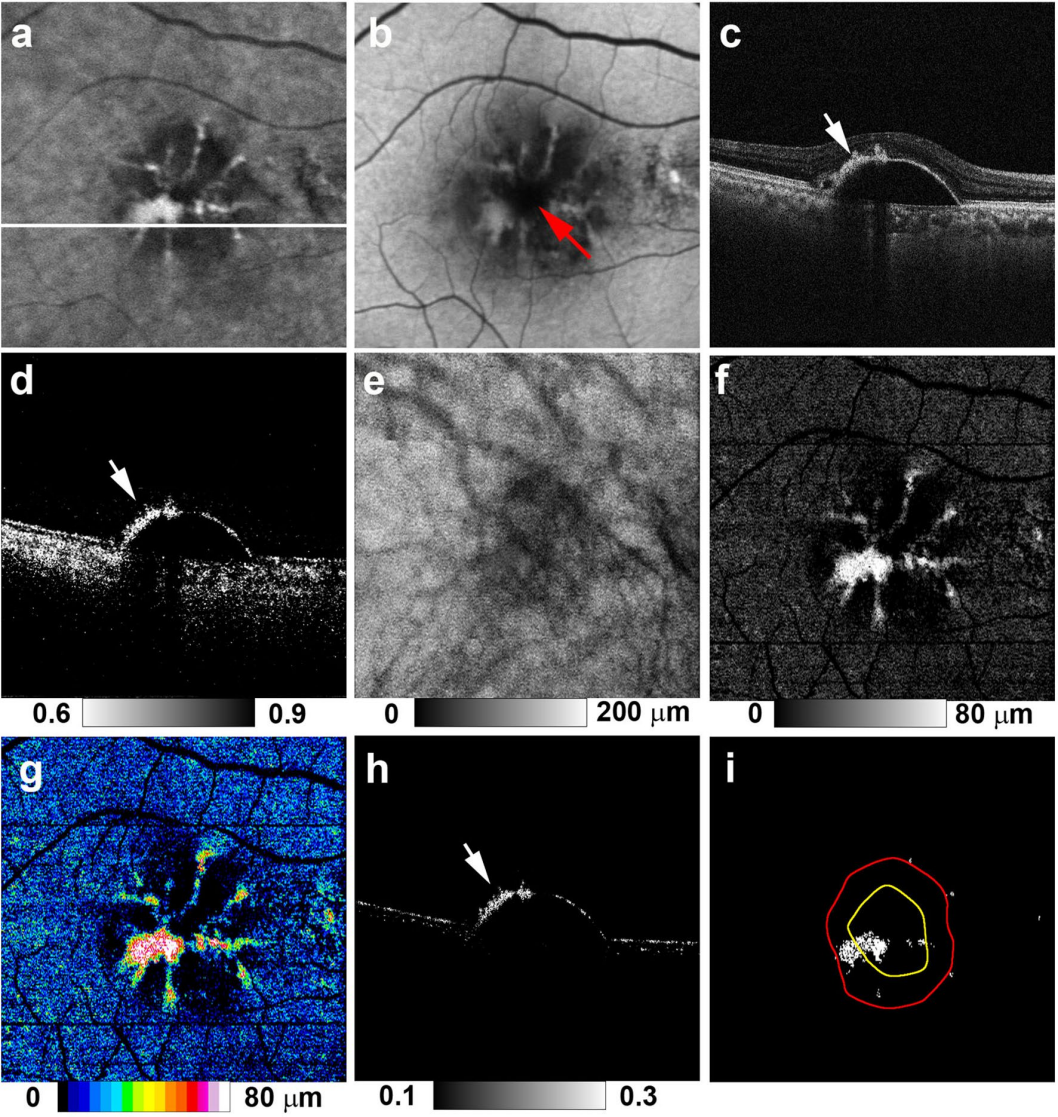
Multimodal imaging of serous PED in the right eye of a 78-year-old man. NIR-AF (**a**) and SW-AF (**b**) images show hyper-AF lesions. In the SW-AF image (**b**), the hyper-AF lesion is obscured in the foveal region (red arrow). The white line in the NIR-AF image (**a**) designates the scanning line of the MC-OCT B-scan images (**c**,**d**,**h**). The standard OCT B-scan image (**c**) shows a thickened RPE band and intraretinal hyper-reflective foci in the hyper-AF lesion. The DOPU B-scan image (**d**) shows melanin accumulation at the RPE band and intraretinal melanin foci (arrow). RPE changes in the hyper-AF lesion are not evident in the chorioretinal melanin thickness map (**e**). The greyscale RPE-melanin thickness map (**f**) is similar to the AF images (**a**,**b**) and RPE-melanin thickness is readily visible on the color-coded RPE-melanin thickness map (**g**). The RPE-melanin B-scan image (**h**) shows RPE-melanin accumulation at the RPE band and intraretinal RPE-melanin foci (arrow). En face distribution of RPE_70_ is shown in panel (**i**). The red line indicates the margin of the PED and the yellow line indicates the axis position for 70% of the maximum PED height. RPE_70_ areas in this patient were distributed in both the peak and slope regions.

**Figure 2:**
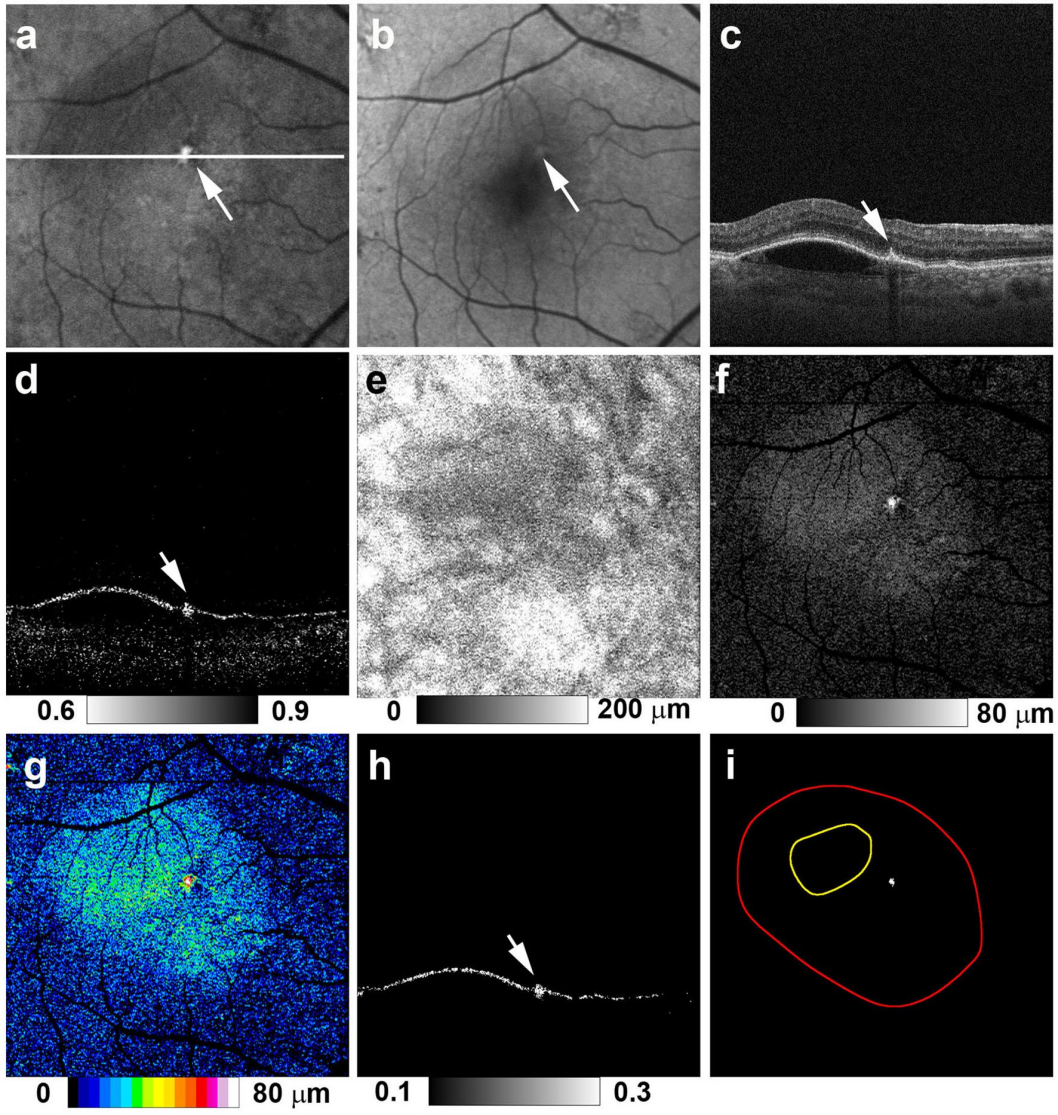
Multimodal imaging of serous PED in the right eye of a 70-year-old man. NIR-AF (**a**) and SW-AF (**b**) images show a hyper-AF lesion (arrows). The white line in image (**a**) designates the scanning line of the MC-OCT B-scan images (**c**,**d**,**h**). The standard OCT B-scan image (**c**) shows a thickened RPE band in the hyper NIR-AF lesion (arrow). The DOPU B-scan image (**d**) shows melanin accumulation at the RPE band (white arrow). RPE changes in the hyper-AF lesion are not evident in the chorioretinal melanin thickness map (**e**). The greyscale RPE-melanin thickness map (**f**) shows similarity with the NIR-AF image (**a**) and RPE-melanin thickness is readily visible in the color-coded RPE-melanin thickness map (**g**). RPE-melanin B-scan image (**h**) shows RPE-melanin accumulation at the RPE band (arrow). *En face* distribution of RPE_70_ is shown in panel (**i**). Red line indicates the margin of the PED and yellow line indicates the axis position for 70% of the maximum PED height. The RPE_70_ area in this patient was solely within the slope region.

NIR-AF imaging showed hyper NIR-AF lesions in the PED (Figs. 1a and 2a) in 23 of 26 eyes examined (88%). SW-AF images showed hyper SW-AF at corresponding locations (Figs. 1b and 2b). However, hyper SW-AF was somewhat obscured in the foveal region, possibly by the presence of macular pigment, in the patient shown in Fig. 1b. MC-OCT provides standard OCT, OCT angiography, and degree of polarization uniformity (DOPU)^30^ data from a single measurement. Standard OCT B-scan images showed a thickened RPE band and intraretinal hyper-reflective foci in hyper NIR-AF lesions (Figs. 1c and 2c). The presence of low DOPU is presumed to indicate depolarization by multiple scattered lights from melanin^22^. A chorioretinal melanin thickness map was computed by counting the number of pixels with low DOPU (< 0.8) on each A-line in the three-dimensional dataset. The resulting chorioretinal melanin thickness map represents the overall thickness of melanin in the choroid and RPE. DOPU B-scan images in polarization-sensitive OCT clearly showed melanin accumulation in the RPE band, as well as intraretinal melanin migration (Figs. 1d and 2d). However, these melanin changes were obscured by choroidal melanin in the chorioretinal melanin thickness maps (Figs. 1e and 2e). For automatic discrimination of RPE-melanin from choroidal melanin based on DOPU images, we computed a new index (F_RPE_) based on the concept of feature engineering by the presence or absence of blood flows^26^. Melanin in both the RPE and choroid showed low DOPU due to depolarization. However, the OCT angiography signal in the RPE cells was low because vascularization was absent, whereas the choroid showed a high OCT angiography signal because of its dense vasculature. The presence of high F_RPE_ is presumed to indicate the presence of RPE-melanin. The RPE-melanin B-scan images were used to determine the distribution of F_RPE_ in the B-scan images, which enabled evaluation of the depth-resolved distribution of RPE-melanin. RPE-melanin thickness maps were created by counting the number of pixels with high F_RPE_ (≥ 0.15) on each A-line in the volume dataset. RPE-melanin thickness maps clearly showed the *en face* distributions of these melanin changes. Greyscale RPE-melanin thickness maps were similar to both NIR-AF and SW-AF images in all patients (Figs. 1f, 2f). Hyper NIR-AF lesions coincided with areas of thickened RPE melanin. This similarity facilitated quantitative evaluation of the RPE-melanin changes in hyper NIR-AF lesions through RPE-melanin thickness maps. The thickness of RPE-melanin, according to location within the PED, could readily be visualized with color-coded RPE-melanin thickness maps (Figs. 1g and 2g). The RPE-melanin B-scan images clearly showed that thickened RPE-melanin lesions were composed of RPE-melanin accumulation at the RPE band and intraretinal RPE-melanin migration (Figs. 1h and 2h). To evaluate the distributions of thickened RPE lesions, the PED area was divided into peak and slope regions. The inner area (70% of the maximum PED height) was defined as the peak region, while the outer residual area of the PED was defined as the slope region (Supplementary Fig. S1 online). Areas of thickened RPE-melanin (RPE_70_: ≥ 70 μm) were distributed in both the peak and slope regions in 19 eyes (Fig. 1i), while they were solely in the slope region in four eyes (Fig. 2i).

For quantitative evaluations, we compared morphometric PED parameters and the parameters associated with RPE_70_ (Table 1). Tomographic volumes in standard OCT images, obtained from MC-OCT, were used to evaluate morphometric parameters (maximum PED height, PED area, PED volume, and slope area ratio). Obliqueness of the slope area was evaluated as the slope area ratio (area of slope region/area of whole PED). A small slope area ratio indicated the presence of a steep slope.

**Table 1:** Morphometric pigment epithelial detachment parameters, RPE_70_ areas, and RPE_70_ area ratios.

Parameter	Mean ± standard deviation (range)
Maximum PED height (mm)	0.288 ± 0.202 (0.015–0.799)
PED area (mm^2^)	7.345 ± 7.378 (0.564–25.294)
PED volume (mm^3^)	1.594 ± 2.333 (0.032–8.718)
Slope area ratio	0.713 ± 0.097 (0.463–0.888)
RPE_70_ area for whole PED (mm^2^)	0.072 ± 0.108 (0.000–0.373)
RPE_70_ area for peak region (mm^2^)	0.020 ± 0.041 (0.000–0.149)
RPE_70_ area for slope region (mm^2^)	0.053 ± 0.080 (0.000–0.341)
RPE_70_ area ratio for whole PED	0.011 ± 0.015 (0.000–0.050)
RPE_70_ area ratio for peak region	0.013 ± 0.033 (0.000–0.150)
RPE_70_ area ratio for slope region	0.011 ± 0.013 (0.000–0.044)

*PED* pigment epithelial detachment, *RPE* retinal pigment epithelium.

**Figure 3:**
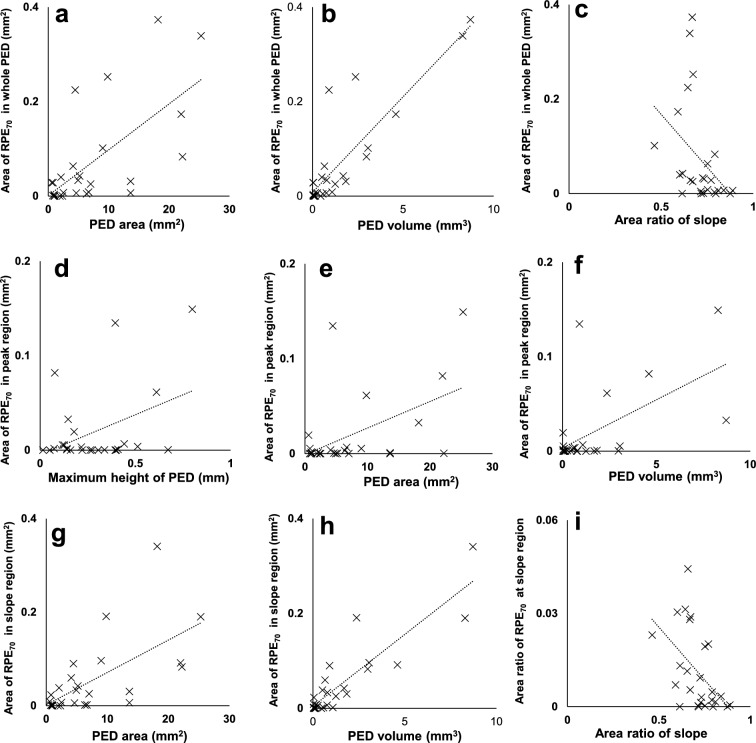
Scatterplots of RPE_70_ areas or area ratios and morphometric PED parameters with statistically significant correlations. Scatterplots of the RPE_70_ area for the whole PED as a function of (**a**) PED area, (**b**) PED volume, and (**c**) slope area ratio. Scatterplots of the RPE_70_ area for the peak region as a function of (**d**) maximum PED height, (**e**) PED area, and (**f**) PED volume. Scatterplots of the RPE_70_ area for the slope region as a function of (**g**) PED area and (**h**) PED volume. (**i**) Scatterplot of the RPE_70_ area ratio for the slope region as a function of slope area ratio.

We calculated the RPE_70_ areas for each whole PED, peak region, and slope region. RPE_70_ areas were significantly greater in the slope region than in the peak region (P = 0.002, Wilcoxon signed-rank test; Table 1). Simple linear regression analysis was performed between RPE_70_ areas and morphometric PED parameters (Table 2, Fig. 3, and Supplementary Fig. S2 online). Figure 3 shows the scatter plots of combinations with statistically significant correlations. The RPE_70_ areas in whole PED, peak region, and slope region showed significant positive correlations with PED area and PED volume. The RPE_70_ area in whole PED showed a significant negative correlation with slope area ratio. The RPE_70_ area in the peak region showed a significant positive correlation with maximum PED height. Subsequently, multiple linear regression analysis was conducted to consider interactions among morphometric parameters. Stepwise multiple linear regression analysis of RPE_70_ area with four morphometric PED parameters (maximum PED height, PED area, PED volume, and slope area ratio) revealed that the RPE_70_ areas in both whole PED and the slope region showed significant positive correlations with PED volume (Table 3).

**Table 2:** Correlations between RPE_70_ areas and morphometric retinal pigment epithelial detachment parameters.

RPE_70_ area	Morphometric PED parameter	Correlation coefficient	P value
RPE_70_ area for whole PED	Maximum PED height	0.368	0.064
	PED area	0.663	< 0.001*
	PED volume	0.873	< 0.001*
	Slope area ratio	− 0.401	0.043*
RPE_70_ area for peak region	Maximum PED height	0.414	0.036*
	PED area	0.500	0.009*
	PED volume	0.577	0.002*
	Slope area ratio	0.320	0.111
RPE_70_ area for slope region	Maximum PED height	0.285	0.158
	PED area	0.640	< 0.001*
	PED volume	0.884	< 0.001*
	Slope area ratio	0.377	0.057

*PED* pigment epithelial detachment, *RPE* retinal pigment epithelium. *Statistically significant correlation.

**Table 3:** Covariance parameter estimates of RPE_70_ areas and morphometric retinal pigment epithelial detachment parameters.

RPE_70_ area	Morphometric PED parameter	β	95% CI	P value
RPE_70_ area for whole PED	Maximum PED height	0.139	− 0.080 to 0.358	0.203
	PED area	− 0.240	− 0.637 to 0.157	0.222
	PED volume	1.005	0.589 to 1.422	< 0.001*
	Slope area ratio	− 0.070	− 0.300 to 0.161	0.537
RPE_70_ area for peak region	Maximum PED height	0.273	− 0.099 to 0.646	0.142
	PED area	0.053	− 0.620 to 0.727	0.871
	PED volume	0.385	− 0.323 to 1.092	0.271
	Slope area ratio	− 0.169	− 0.560 to 0.222	0.379
RPE_70_ area for slope region	Maximum PED height	0.047	− 0.161 to 0.255	0.645
	PED area	− 0.353	− 0.729 to 0.023	0.065
	PED volume	− 0.163	0.768 to 1.558	< 0.001*
	Slope area ratio	− 0.007	− 0.226 to 0.211	0.948

*CI* confidence interval, *PED* pigment epithelial detachment, *RPE* retinal pigment epithelium. *Statistically significant.

To eliminate the contributing factor of area, the RPE_70_ area ratios (RPE_70_ area in whole PED/area of whole PED, RPE_70_ area in peak region/area of peak region, RPE_70_ area in slope region/area of slope region) were calculated. First, simple linear regression analysis was performed between RPE_70_ area ratios and morphometric PED parameters (Table 4, Fig. 3, and Supplementary Fig. S3 online). The RPE_70_ area ratio in the slope region showed a significant negative correlation with the slope area ratio (Table 4, Fig. 3). Stepwise multiple linear regression analysis confirmed this significant negative correlation (Table 5).

**Table 4:** Correlations between RPE_70_ area ratios and morphometric retinal pigment epithelial detachment parameters.

RPE_70_ area ratio	Morphometric PED parameter	Correlation coefficient	P value
RPE_70_ area ratio for whole PED	Maximum PED height	0.081	0.693
	PED area	− 0.080	0.696
	PED volume	0.092	0.656
	Slope area ratio	− 0.286	0.156
RPE_70_ area ratio for peak region	Maximum PED height	0.009	0.967
	PED area	− 0.152	0.459
	PED volume	− 0.077	0707
	Slope area ratio	0.012	0.955
RPE_70_ area ratio for slope region	Maximum PED height	0.061	0.767
	PED area	− 0.010	0.963
	PED volume	0.224	0.270
	Slope area ratio	0.511	0.008*

*PED* pigment epithelial detachment, *RPE* retinal pigment epithelium. *Statistically significant correlation.

**Table 5:** Covariance parameter estimates of RPE_70_ area ratios and morphometric pigment epithelial detachment parameters.

RPE_70_ area ratio	Morphometric PED parameter	β	95% CI	P value
RPE_70_ area ratio for whole PED	Maximum PED height	0.135	− 0.316 to 0.585	0.541
	PED area	− 0.457	− 1.272 to 0.358	0.257
	PED volume	0.348	− 0.508 to 1.204	0.404
	Slope area ratio	− 0.230	− 0.703 to 0.243	0.323
RPE_70_ area ratio for peak region	Maximum PED height	0.070	− 0.409 to 0.548	0.765
	PED area	− 0.315	− 1.181 to 0.551	0.458
	PED volume	0.165	− 0.744 to 1.074	0.710
	Slope area ratio	− 0.001	− 0.503 to 0.502	0.998
RPE_70_ area ratio for slope region	Maximum PED height	0.092	− 0.301 to 0.492	0.636
	PED area	− 0.481	− 1.204 to 0.242	0.181
	PED volume	0.444	− 0.316 to 1.203	0.238
	Slope area ratio	− 0.424	− 0.844 to − 0.043	0.048*

*CI* confidence interval, *PED* pigment epithelial detachment, *RPE* retinal pigment epithelium. *Statistically significant correlation.

## Discussion

In the present study, we evaluated the RPE changes in serous PEDs by means of MC-OCT imaging. Multimodal imaging analysis with RPE-melanin imaging by MC-OCT can provide new insights concerning clinical AF findings. Both NIR-AF and RPE-melanin imaging by MC-OCT are sensitive to melanin associated with the RPE^14,26^. The similarities between NIR-AF and RPE-melanin thickness maps demonstrate the complementary nature of these imaging techniques. RPE-melanin imaging with MC-OCT provides three-dimensional information about RPE-melanin that cannot be obtained by NIR-AF imaging. RPE-melanin B-scan OCT images showed intraretinal RPE-melanin migration and RPE-melanin accumulation at the RPE-Bruch’s membrane band in hyper NIR-AF lesions. Concomitant conformations of hyper SW-AF in these lesions indicate that the RPE changes are the most likely origin of these melanin-related findings; these changes include intraretinal RPE migration, stacked RPE cells, and RPE dysmorphia^6–8^. Notably, these RPE changes have been suggested to represent RPE activity^7^; our multimodal imaging technique might be useful in the evaluation of the RPE cellular activity in PEDs.

The present study showed positive correlations of PED volume with the area of active RPE lesions (RPE_70_) in both the whole PED and the slope region (Table 3). This finding is consistent with the results of our previous polarization-sensitive OCT study regarding serous PEDs^8^. A study with drusenoid PED also confirmed the correlation with PED volume^7^. Hypoxia may contribute to RPE activation, due to the reduction of oxygen distribution from the choriocapillaris^31,32^. The diffusion of oxygen to the RPE could be impaired by the increased distance from the choroid in large PEDs^7^. If hypoxia is a predominant contributing factor with respect to RPE activation, RPE changes should occur mainly in the peak regions of PEDs, at a specific distance from the choroid.

However, RPE changes frequently occurred in the slope regions of serous PEDs. In the present study, RPE_70_ existed in the slope region in 23 of 26 eyes (88%); the RPE_70_ area was significantly greater in the slope region than in the peak region. This frequent occurrence of RPE changes in the slope region was also reported in drusenoid PEDs^6^, despite a possibly larger contribution of hypoxia with the presence of drusenoid material. The development of RPE activation in the slope region suggests the presence of induction factors other than hypoxia. One possible explanation involves size: there was a close relationship between the RPE_70_ area and the PED area, according to simple linear regression analysis (Table 2). The RPE_70_ area might increase along with the underlying PED area. To eliminate the contributing factor of area, we evaluated the RPE_70_ area ratio. We found a negative correlation between the slope area ratio and the RPE_70_ area ratio in the slope region. A small slope area ratio indicated the presence of a steep slope; thus, active RPE lesions in the slope region tended to occur with steep slopes. A possible predisposing factor for a steep slope involves mechanical tension due to architectural changes in the RPE band. Mechanical forces may contribute to remodeling of the extracellular matrix around RPE cells and may facilitate the migration of RPE cells^33^. Although the exact mechanism by which RPE migration occurs in serous PEDs is not yet fully understood, hypoxia and mechanical stress are possible causative factors in RPE changes.

There were several limitations to this study. First, because it involved a relatively small number of patients without follow-up assessments, this preliminary study evaluated only some aspects of RPE changes in serous PEDs. Further studies, involving many patients with follow-up data, are needed to evaluate RPE changes in greater detail. Second, RPE-melanin measurement with MC-OCT in this study was based on depolarization by multiple scattered lights. Depolarization could be induced by hard exudate without blood flow signals^34^; hence, it was difficult to distinguish RPE-melanin from hard exudates when using MC-OCT imaging. To avoid the possible presence of hard exudates, we excluded patients with neovascular PED from this study. An evaluation of other subtypes of AMD, including neovascular PED, is important for the comprehensive investigation of RPE damage in patients with AMD. Further development of the imaging algorithm is important for a wide range of applications that involve RPE-melanin measurement. Third, the multimodal imaging approach of this study did not include fluorescein angiography or indocyanine green angiography. These angiography imaging techniques might provide additional information about RPE changes^18,35^. Fourth, the detection of intraretinal RPE changes in the present study, based on the presence of melanin and lipofuscin, was limited by the possible presence of infiltrating inflammatory cells^36^. If inflammatory cells ingested melanosomes and lipofuscin from disintegrating RPE cells, our imaging system might have misidentified those inflammatory cells as RPE cells. Additional histopathological studies are needed to address this limitation. Fifth, morphometric PED parameters were calculated through manual segmentation in this study. The automatic segmentation of RPE lines in diseased retina was frequently problematic because of the erroneous identification of the RPE line and Bruch’s membrane^37^. Further development of imaging techniques is necessary for clinical applications that rely on automated calculation. Sixth, although a previous study showed a monotonic relationship between DOPU and melanin, measurement of the RPE-melanin thickness could be affected by the DOPU kernel size or melanin packing density in RPE cells. Therefore, RPE-melanin thickness maps do not represent the actual thickness of RPE-melanin, although they are proportional to the thickness of the RPE-melanin. Seventh, a threshold of F_RPE_ (≥ 0.15) in MC-OCT imaging was used to calculate the area of RPE melanin defects, in accordance with the method used in our previous studies^9,25,26^. With this threshold, the RPE-melanin line at the normal RPE band occasionally became unclear (Fig. 1h). Finally, we used a cutoff value of ≥ 70 μm for statistical analysis of the areas of thickened RPE-melanin. Further analysis with a larger number of patients is necessary to determine the appropriate evaluation threshold.

In conclusion, this study demonstrated the clinical utility of multimodal imaging with RPE-melanin OCT imaging and MC-OCT for evaluation of RPE changes in serous PEDs. MC-OCT imaging enables three-dimensional evaluation of AF images; the combination of MC-OCT and AF imaging could be useful for assessment of various macular diseases. MC-OCT is a promising tool as a next-generation imaging system for the clinical evaluation of macular diseases.

## Methods

### Patients

We prospectively examined 26 eyes of 21 Japanese patients with serous PEDs due to AMD (13 men, 8 women; age range, 55–83 years; mean age, 72.1 years). Among them, three patients were treated for hypertension, one patient was treated for diabetes, and one patient was treated for both hypertension and diabetes. To exclude the influence of these systemic diseases, eyes with retinal diseases other than AMD were excluded. Patients were considered to have “serous PED” when they exhibited serous PED without retinal hemorrhage, hard exudates, or choroidal neovascularization. OCT angiography images obtained from commercial OCT (DRI-OCT Triton, Topcon, Tokyo, Japan) and MC-OCT were used to exclude eyes with choroidal neovascularization. To avoid the possible influence of treatments for AMD, eyes with prior intravitreal injection or laser treatment were excluded. Eyes with severe cataract or other eye diseases that could significantly compromise the image quality were also excluded from this study.

This prospective, observational, cross-sectional study followed the tenets of the Declaration of Helsinki, and Institutional Review Board approval was obtained from the Tokyo Medical University Institutional Review Board (IB-1615 and T2019-0072). The study was registered in a public database (UMIN000026307 and 000039648; http://www.umin.ac.jp/ctr/index-j.htm). Written informed consent was obtained from each participant before any study procedures or examinations were performed.

### MC-OCT system

A detailed description of the prototype Jones-matrix MC-OCT has been published^28^. MC-OCT provides standard OCT, OCT angiography, and DOPU data from a single measurement. Standard OCT images were obtained by coherent composition of four repetitive scans^28^. OCT angiography was calculated using the complex Jones matrix correlation method with noise correction^38^. DOPU was calculated with Makita’s noise correction using a 3 × 3 pixel kernel^39^. The light source was a swept-source laser with a central wavelength of 1050 nm. The axial scan speed was 100,000 A-scans/s and the depth resolution in tissue was 6.0 μm. A horizontal-fast raster scanning protocol with 512 A-lines × 256 B-scans was used for volumetric scans; the approximate measurement area on the retina was 6.0 × 6.0 mm. For quantitative measurements, the transverse magnification of MC-OCT images was calibrated using a modified Littman’s method^40^. B-scan measurements were repeated four times at a single location; the acquisition duration for volumetric measurement was 6.6 s. MC-OCT volumes without significant motion artifacts were used for this study.

### Morphometric PED parameters

Tomographic volumes in standard OCT images, obtained from MC-OCT, were used for morphometric evaluation of serous PEDs using FIJI image-processing software^41^.

1. PED area: The locations of the PED margins in *en face* OCT images were manually determined using series of B-scan images. PED area was calculated as the transverse inner area within the margin of the PED.
2. Maximum PED height was measured as the greatest distance between Bruch’s membrane and the outer boundary of the RPE band.
3. PED volume: The inner boundary of the PED was manually segmented in each B-scan image. The total PED volume was determined by summing the volumes of individual segments using the Cavalieri principle of stereological analysis ^42^.
4. Peak and slope regions: Transverse locations with a height equal to 70% of the maximum PED height were manually determined using series of B-scan images (Supplementary Fig. S1a online). In *en face* OCT images, the inner area (70% of the maximum PED height) was defined as the peak region, while the outer residual area of the PED was defined as the slope region (Supplementary Fig. S1b online).

### RPE-melanin-specific contrast OCT imaging

For automatic discrimination of RPE-melanin from choroidal melanin based on DOPU images, a new index (F_RPE_) was computed using an attenuation coefficient^43^, the DOPU, and the blood flow signal in OCT angiography, as follows^26^:

$$ {\text{F}}_{{{\text{RPE}}}} = {\text{attenuation}}\;{\text{coefficient}} \times (1{-}{\text{DOPU}}) \times (1{-}{\text{OCTA}}_{{\text{b}}} ), $$

where OCTA_b_ is the binarized OCT angiography signal. This index was computed to selectively represent RPE-melanin by the absence of blood flow in the RPE. The RPE-melanin B-scan images were computed as the distribution of F_RPE_ in the B-scan images; this enabled evaluation of the depth-resolved distribution of RPE-melanin. RPE-melanin thickness maps were created by counting the number of pixels with high F_RPE_ (≥ 0.15) on each A-line in the volume dataset. The area of thickened RPE-melanin (RPE_70_: ≥ 70 μm) was calculated from RPE-melanin thickness maps using image-processing software (FIJI)^41^ (Fig. 3c).

### Multimodal imaging

RPE-melanin OCT images were compared with NIR-AF and SW-AF images. Both NIR-AF images (785-nm excitation, emission > 800 nm) and SW-AF images (488-nm excitation, emission > 500 nm) were obtained using an HRA2 (Heidelberg Engineering, Heidelberg, Germany), and were saved in eight-bit greyscale images. For comparison of AF images and MC-OCT images, retinal vascular architecture was manually aligned across images using image processing software (Adobe Photoshop CS5, Adobe Systems, San Jose, CA, USA). A retina specialist (T.I.) subjectively evaluated the distribution of hyper-AF lesions.

## Supplementary Information

Supplementary Information.
