# Supplementary material for: Evaluation of retinal pigment epithelium changes in serous pigment epithelial detachment in age-related macular degeneration
Source: Sci Rep. 2021 Feb 2;11:2764. doi: 10.1038/s41598-021-82563-z (PMC7854605; doi:10.1038/s41598-021-82563-z)
Supplement: Supplementary file 1 — Supplementary Information. [file 41598_2021_82563_MOESM1_ESM.pdf]

Evaluation of retinal pigment epithelium changes in serous pigment epithelial detachment in age-related macular degeneration

Masahiro Miura, Shuichi Makita, Yoshiaki Yasuno, Takuya Iwasaki, Shinnosuke Azuma, Toshihiro Mino, Tatsuo Yamaguchi

Supplementary Figure S1

Methods for measurement of RPE_{70} areas for the peak and slope regions.

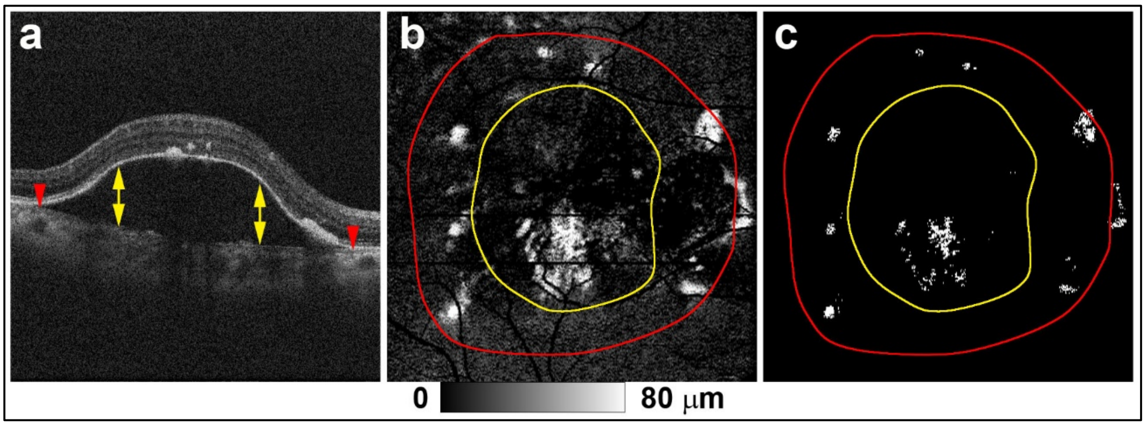

A standard optical coherence tomography (OCT) B-scan image (a) was used to determine the margins of the PED (red arrowheads) and the axis positions for 70% of the maximum PED height (yellow arrows). RPE-melanin thickness map is shown in panel b and *en face* map of RPE_{70} is shown in panel c. Red lines indicate the margin of the PED and yellow lines indicate the axis positions for 70% of the maximum PED height. The area within the yellow line was defined as the peak region, while the area between the red and yellow lines was defined as the slope region. The *en face* map of RPE_{70} (c) was used to measure the RPE_{70} area and RPE_{70} area ratio for the whole PED, peak region, and slope region.

Supplementary Figure S2

Scatterplots of RPE_{70} areas and morphometric PED parameters without statistically significant correlations.

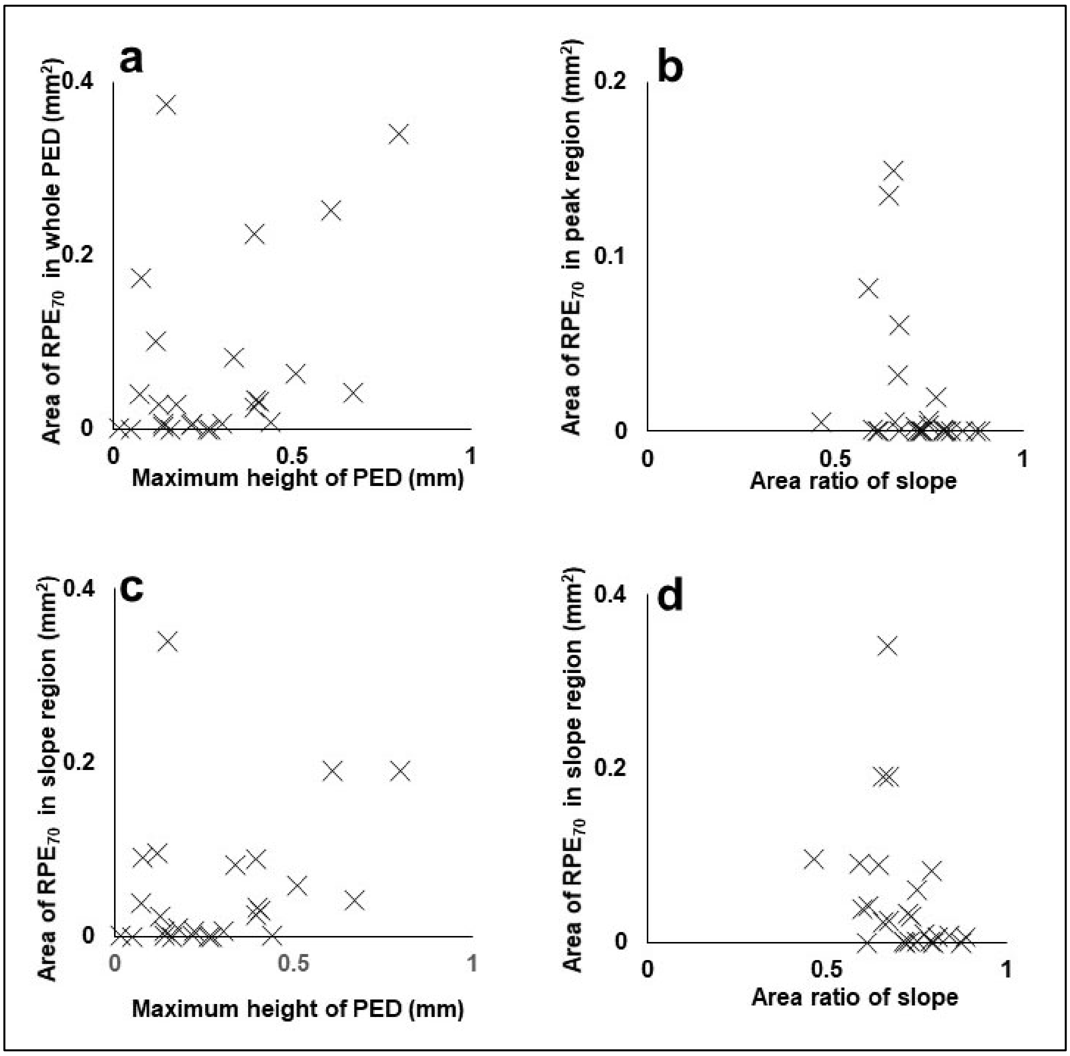

(a) Scatterplot of the RPE_{70} area for the whole PED as a function of maximum PED height. (b) Scatterplot of the RPE_{70} area for the peak region as a function of slope area ratio. Scatterplots of the RPE_{70} area for the slope region as a function of (c) maximum PED height of PED and (d) area ratio.

Supplementary Figure S3

Scatterplots of RPE$_{70}$ area ratios and morphometric PED parameters without statistically significant correlations.

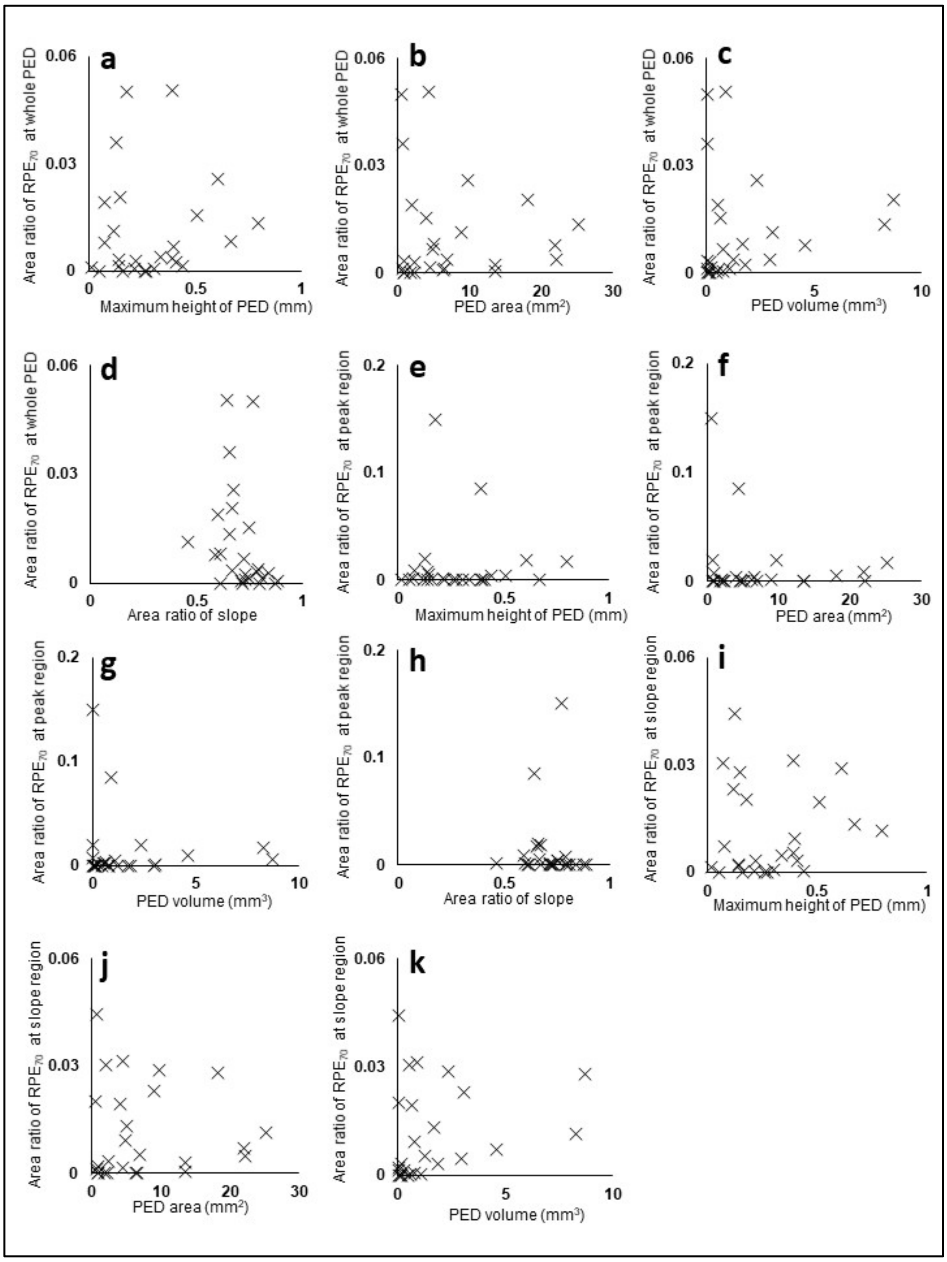

Scatterplots of RPE$_{70}$ area ratios for the whole PED as a function of (a) maximum PED height, (b) PED area, (c) PED volume, and (d) slope area ratio. Scatterplots of the area ratio of RPE$_{70}$ for the peak region as a function of (e) maximum PED height, (f) PED area, (g) PED volume, and (h) slope area ratio. Scatterplots of the RPE$_{70}$ area ratio for the slope region as a function of (i) maximum PED height, (j) PED area, and (k) PED volume.

Morphometric PED parameters, RPE_{70} areas, and RPE_{70} area ratios

Patient	Eye	Max height (mm)	PED area (mm^2)	PED volume (mm^3)	Slope area ratio	RPE_{70} area (mm^2) Whole PED	RPE_{70} area (mm^2) Peak region	RPE_{70} area (mm^2) Slope region	RPE_{70} area ratio Whole PED	RPE_{70} area ratio Peak region	RPE_{70} area ratio Slope region
1	R	0.39	4.45	0.91	0.64	0.2244	0.1346	0.0898	0.0505	0.0851	0.0313
2	R	0.31	13.62	0.77	0.89	0.0064	0.0000	0.0064	0.0005	0.0000	0.0005
2	L	0.41	13.62	1.84	0.73	0.0311	0.0008	0.0303	0.0023	0.0002	0.0030
3	R	0.12	9.00	3.06	0.46	0.1015	0.0053	0.0962	0.0113	0.0011	0.0231
4	R	0.08	22.00	4.60	0.59	0.1733	0.0818	0.0914	0.0079	0.0091	0.0070
5	R	0.02	0.75	0.03	0.73	0.0008	0.0000	0.0008	0.0010	0.0000	0.0014
5	L	0.05	0.97	0.15	0.61	0.0000	0.0000	0.0000	0.0000	0.0000	0.0000
6	L	0.22	6.34	0.57	0.71	0.0046	0.0032	0.0013	0.0007	0.0018	0.0003
7	R	0.15	18.17	8.72	0.67	0.3733	0.0326	0.3406	0.0205	0.0054	0.0281
8	R	0.61	9.82	2.37	0.67	0.2523	0.0613	0.1910	0.0257	0.0191	0.0289
8	L	0.14	4.62	0.27	0.81	0.0063	0.0005	0.0058	0.0014	0.0006	0.0015
9	R	0.80	25.29	8.29	0.65	0.3392	0.1491	0.1900	0.0134	0.0171	0.0115
9	L	0.34	22.22	2.98	0.79	0.0831	0.0003	0.0829	0.0037	0.0001	0.0047
10	L	0.16	1.92	0.06	0.87	0.0000	0.0000	0.0000	0.0000	0.0000	0.0000
11	R	0.18	0.56	0.03	0.77	0.0282	0.0194	0.0088	0.0500	0.1500	0.0202
12	R	0.13	0.78	0.05	0.66	0.0282	0.0053	0.0229	0.0360	0.0198	0.0443
12	L	0.22	2.50	0.16	0.84	0.0070	0.0000	0.0070	0.0028	0.0000	0.0033
13	L	0.26	1.05	0.11	0.72	0.0000	0.0000	0.0000	0.0000	0.0000	0.0000
14	R	0.27	2.33	0.45	0.80	0.0000	0.0000	0.0000	0.0000	0.0000	0.0000
15	R	0.67	5.18	1.69	0.62	0.0424	0.0003	0.0421	0.0082	0.0001	0.0132
16	R	0.51	4.09	0.66	0.75	0.0632	0.0037	0.0595	0.0155	0.0036	0.0194
17	L	0.40	4.92	0.79	0.72	0.0332	0.0000	0.0332	0.0068	0.0000	0.0093
18	R	0.07	2.09	0.51	0.60	0.0397	0.0015	0.0382	0.0190	0.0019	0.0304
19	R	0.39	7.01	1.25	0.67	0.0255	0.0000	0.0255	0.0036	0.0000	0.0054
19	L	0.44	6.67	1.08	0.75	0.0082	0.0066	0.0016	0.0012	0.0040	0.0003
20	L	0.14	0.97	0.04	0.79	0.0031	0.0015	0.0016	0.0032	0.0073	0.0021

L: left, PED: pigment epithelial detachment, R: right, RPE: retinal pigment epithelium
